# Tuberculosis Among Artisanal Miners in the Mulongo Health Zone, Democratic Republic of Congo: Unmatched Case-Control Study

**DOI:** 10.2196/93635

**Published:** 2026-10-19

**Authors:** Josué Mutangala Muloye, Fiston Ilunga Mbayo, Jacques Muteba Kakoma, François Kitobo Mashika, Jean Claude Banze Kabwe Ilunga, Pacifique Kanku Wa Ilunga, Gloire Kasongo Lupitshi, Didy Lwamba Kabiya, Paulin Beya Wa Bitadi Mutombo

**Affiliations:** 1 Health Zone of Lwamba Lwamba, Haut Lomami The Democratic Republic of The Congo; 2 School of Public Health University of Malemba Nkulu Malemba Nkulu, Haut Lomami The Democratic Republic of The Congo; 3 General Reference Hospital of Malemba Nkulu Malemba Nkulu, Haut Lomami The Democratic Republic of The Congo; 4 University of Kamina Kamina, Haut-Lomami The Democratic Republic of The Congo; 5 Provincial Health Division of Haut-Lomami Kamina, Haut Lomami The Democratic Republic of The Congo; 6 Higher Institute of Medical Techniques of Kamina Kamina, Haut Lomami The Democratic Republic of The Congo; 7 School of Public Health Medicine University of Kinshasa Kinshasa, Kinshasa The Democratic Republic of The Congo

**Keywords:** tuberculosis, artisanal miners, poverty, HIV, overcrowding, Democratic Republic of Congo, case-control study

## Abstract

**Background:**

Tuberculosis (TB) remains a major global health challenge, with 10.6 million new cases and 1.3 million deaths reported in 2022. Artisanal miners in sub‑Saharan Africa are particularly vulnerable due to poverty, overcrowded living conditions, and high HIV prevalence. In the Democratic Republic of Congo (DRC), TB prevalence among miners is high, yet determinants remain insufficiently studied.

**Objective:**

This study aimed to identify sociodemographic, behavioral, and clinical factors associated with TB among artisanal miners in the Mulongo health zone, Haut‑Lomami Province, DRC.

**Methods:**

We conducted an unmatched case-control study among miners aged ≥15 years. Data were collected using a validated questionnaire covering socioeconomic, behavioral, and biomedical variables. Interviewers were blinded to TB status to minimize bias. Determinants were assessed using bivariate and multivariable logistic regression.

**Results:**

Poverty was strongly associated with TB (adjusted odds ratio [aOR] 3.16, 95% CI 1.95-5.11; *P*<.001). Primary education was associated with increased TB risk (aOR 2.78, 95% CI 1.04-7.44; *P*=.04), while secondary education showed a borderline association (aOR 2.53, 95% CI 0.93-6.89; *P*=.07). Higher education was protective (aOR 0.62, 95% CI 0.40-0.95; *P*=.03). Poor knowledge of TB transmission (aOR 2.11, 95% CI 1.16-3.83; *P*=.02), cause (aOR 1.79, 95% CI 1.00-3.20; *P*=.049), and diagnosis (aOR 2.49, 95% CI 1.48-4.17; *P*=.001) were consistently linked to higher vulnerability. HIV infection was significantly associated with TB (aOR 4.65, 95% CI 1.00-21.74; *P*=.049). Overcrowding was more frequent among TB cases, but in adjusted models, the association was borderline (aOR 1.65, 95% CI 0.95-2.85; *P*=.07). Tobacco use (aOR 1.47, 95% CI 0.83-2.61; *P*=.18), alcohol consumption (aOR 1.18, 95% CI 0.70-1.98; *P*=.54), and nutritional status (aOR 1.36, 95% CI 0.78-2.37; *P*=.28) were not significantly associated with TB.

**Conclusions:**

TB among artisanal miners appears to be driven primarily by poverty, limited education, poor knowledge, and HIV infection. Overcrowding showed a suggestive but nonsignificant association. Integrated interventions should prioritize poverty alleviation, improved housing, tailored health education, and strengthened TB and HIV services, while further studies are needed to clarify the role of overcrowding and other contextual factors.

## Introduction

Tuberculosis (TB) remains one of the leading infectious diseases worldwide, with significant morbidity and mortality, particularly in low‑ and middle‑income countries. According to the World Health Organization (WHO), approximately 10.6 million people developed TB in 2022, and 1.3 million died from the disease, making it a persistent global health challenge [1]. Transmission occurs primarily through airborne droplets, but occupational and environmental exposures can substantially increase the risk of infection [2].

Health care workers and miners are among the most vulnerable populations due to repeated exposure to infectious aerosols and poor working conditions [3,4]. In sub‑Saharan Africa, artisanal miners face compounded risks: overcrowded living environments, limited access to health services, and a high prevalence of HIV, which further exacerbates susceptibility to TB [5,6]. Studies from Cameroon, Morocco, India, Ethiopia, Nigeria, and the Democratic Republic of Congo (DRC) have consistently reported TB prevalence rates among miners ranging from 31% to more than 70%, underscoring the magnitude of the problem [7‑12].

In the DRC, TB remains a major public health issue, with mining regions such as Haut‑Lomami particularly affected. Recent studies in Lubumbashi and Kalemie reported TB prevalence among miners exceeding 60%, highlighting the urgent need for targeted interventions [7,8]. Contributing factors include inadequate training on infection prevention, lack of personal protective equipment, absence of infection control committees, and systemic underreporting [9,10]. In contrast, high‑income countries have implemented robust surveillance systems and continuous training programs, resulting in lower occupational TB incidence [11].

Overcrowding (defined as ≥3 persons per room) is widely recognized as a determinant of TB transmission in mining communities. Our study specifically assessed this factor alongside poverty, education, knowledge, and HIV infection. Despite the recognized burden, few studies have comprehensively examined the determinants of TB among artisanal miners in the DRC, leaving a critical gap in evidence to guide interventions. Addressing this gap is essential to inform occupational health policies and strengthen TB control in mining communities. Furthermore, this research aligns with the WHO End TB Strategy, which aims to reduce TB incidence and mortality by 90% by 2030 [12].

In the Mulongo health zone, artisanal mining is a major livelihood, and adolescents as young as 15 years are actively engaged in mining activities. Including participants aged 15 years and older was therefore essential to capture the demographic reality of miners in this region. Most miners typically fall within the 15- to 44-year age range, reflecting both early entry into mining and sustained adult participation. Knowledge gaps regarding TB transmission, symptoms, and diagnosis have been documented in similar mining settings [5,13], underscoring the importance of assessing awareness levels in this population. To ensure methodological rigor, the study used a validated questionnaire adapted from WHO Infection Prevention and Control Assessment Tool–Minimum Requirements (IPCAT-MR) [1] and the Centers for Disease Control and Prevention (CDC) Infection Control Assessment and Response (ICAR) [2] tools, with good internal consistency (Cronbach α=0.82). Therefore, this study aimed to identify the factors associated with TB among artisanal miners in the Mulongo health zone, Haut‑Lomami Province. By focusing on sociodemographic, behavioral, and clinical determinants, the findings are expected to contribute to improved TB control strategies in mining communities and support global efforts to end TB.

## Methods

### Study Design and Setting

We conducted an unmatched case-control study between January 2020 and December 2022 in the Mulongo health zone, Haut‑Lomami Province, DRC. This design was chosen because it is suitable in a mining context where the population is well defined but difficult to follow longitudinally. It allows the identification of factors associated with TB by comparing miners with and without the disease recruited from the same environment. A prospective cohort or trial was not feasible due to the high mobility of miners, logistical constraints, and limited resources. The findings should therefore be interpreted as associations rather than causal relationships. It is also important to emphasize that this design is observational in nature and does not establish temporality between exposure and disease occurrence, which limits the ability to infer causality [14,15].

### Study Population and Recruitment

The study population consisted of artisanal miners actively working in the Mulongo health zone. Cases were defined as miners newly diagnosed with pulmonary TB according to WHO guidelines, confirmed either bacteriologically (GeneXpert or microscopy) or clinically validated by a physician, and recorded in local TB registers. Controls were miners without TB, confirmed by the absence of a diagnosis in TB registers and by systematic clinical examination.

Cases and controls were recruited simultaneously from the same mining sites and during the same period to minimize selection bias. The sampling frame was exhaustive, as all eligible miners identified across the 7 mining sites were directly contacted by trained field investigators. In total, 320 miners met the eligibility criteria; 95.3% (n=305) agreed to participate, and 3.1% (n=10) were excluded due to incomplete data, resulting in a final analytical sample of 295 (92.2%) miners. The 25 nonparticipants included 15 refusals (4.7%) and 10 exclusions (3.1%). Nonparticipation was mainly due to personal constraints or lack of availability and was not related to TB status.

Inclusion criteria required a minimum age of 15 years, permanent residence in the Mulongo health zone, and provision of written informed consent. Miners who were nonresidents, had incomplete medical records, or declined participation were excluded. The age group of 15-24 years included both adolescents (15-17 years) and young adults (18-24 years), reflecting the demographic composition of the mining sites.

### Age of Participants

Participants aged 15 years and older were included in the study. This threshold was chosen because artisanal mining in the Mulongo health zone typically involves adolescents as young as 15 years old, who actively participate in mining activities alongside adults. Including this age group ensured that the study captured the full demographic composition of miners in the area. Most miners in this region are aged between 15 and 44 years, reflecting both early entry into mining and sustained adult participation. This age distribution is consistent with local demographic data and provides a representative picture of the mining workforce.

### Sample Size Calculation

The sample size was calculated using Schlesselman formula for case-control studies [16,17]:

n = ((Zα/2 + Zβ)2 × [p1(1 − p1) + p2(1 − p2)]) / (p1 − p2)

where α=5%, power=80%, p1 was the expected exposure proportion among controls, and p2 was the expected exposure proportion among cases. The calculation yielded a minimum required sample size of 270 participants. The final sample included 295 miners (n=125, 42.4% cases and n=170, 57.6% controls), which exceeded the minimum requirement and ensured a statistical power of approximately 82%, thereby providing sufficient precision to detect the associations of interest.

### Data Collection Procedures

Data were collected using a structured questionnaire administered face‑to‑face by trained interviewers. The questionnaire covered sociodemographic characteristics, knowledge about TB, health behaviors, and health status. Daily expenditures were reported in Congolese francs, converted into US dollars, and classified as “acceptable” if equal to or above US $2 per day (international poverty line) and “unacceptable” if below this threshold. Living conditions were considered overcrowded when at least 3 individuals shared a single bedroom.

Knowledge about TB was assessed through questions on transmission, cause, and diagnosis; good knowledge was defined as providing correct answers to most items. Alcohol and tobacco consumption were self‑reported, with a distinction between regular use and nonuse. Medical records were reviewed to confirm TB and HIV diagnoses. Anthropometric measurements were taken to calculate BMI, categorized according to WHO standards: undernutrition (<18.5 kg/m^2^), normal (18.5-24.9 kg/m^2^), overweight (25-29.9 kg/m^2^), and obesity (≥30 kg/m^2^).

### Information Bias Control

To minimize information bias, interviewers were blinded to the TB status of participants during questionnaire administration. Field investigators received standardized training and applied uniform procedures for all miners, regardless of case or control status. This ensured that responses were collected objectively and reduced the risk of differential probing, interviewer bias, or inconsistent data collection.

### Instrument Validation

The questionnaire was pretested with 20 artisanal miners in a neighboring health zone to assess clarity, cultural appropriateness, and relevance of the items. On the basis of feedback, minor linguistic adjustments were made, notably replacing the term “promiscuity” with “overcrowding” to better reflect local terminology. Internal consistency was evaluated using Cronbach α, which yielded a coefficient of 0.82, indicating good reliability [18,19].

The final version of the questionnaire is provided in Multimedia Appendix 1 to facilitate transparency and reproducibility.

### Variables and Operational Definitions

The dependent variable was TB status (case vs control). Independent variables included age, educational level, daily expenditures, overcrowding, TB knowledge, alcohol and tobacco use, HIV status, diabetes, and nutritional status. All independent variables were coded as binary or categorical, with explicit identification of reference categories in the statistical models.

Daily household expenditures were reported in Congolese francs, converted into US dollars, and coded as 0=acceptable (≥US $2/day) and 1=unacceptable (<US $2/day), with “acceptable” as the reference category. Overcrowding was defined as ≥3 persons per room and coded as 1=yes (risk exposure) and 0=no (<3 persons per room), with “no overcrowding” as the reference category.

Educational level was categorized as none, primary, secondary, and higher education or university, with “higher education or university” as the reference category. TB knowledge was assessed through items on transmission, cause, and diagnosis, and coded as 0=good knowledge (reference) and 1=poor knowledge, based on correct responses to more than 50% of items. An example question was “Is tuberculosis transmitted through the air?” Thus, an odds ratio (OR) greater than 1 indicates that poor knowledge is associated with an increased risk of TB, while an OR less than 1 suggests an apparent protective effect that requires cautious interpretation. Alcohol and tobacco use were coded as 0=no and 1=yes, with “no use” as the reference category. HIV status was confirmed by rapid testing and was coded as 0=negative and 1=positive, with “negative” as the reference category. Diabetes was identified through medical history or fasting blood glucose ≥126 mg/dL and was coded as 0=no and 1=yes, with “no diabetes” as the reference category. Nutritional status was assessed by BMI and categorized according to WHO standards: undernutrition (<18.5 kg/m^2^), normal (18.5-24.9 kg/m^2^), overweight (25-29.9 kg/m^2^), and obesity (≥30 kg/m^2^) [25‑30].

### Handling of Missing Data

Incomplete questionnaires were excluded at the point of data collection. The overall proportion of missing data was 3.4%, primarily affecting alcohol and tobacco variables. Overcrowding data were complete for all participants and therefore fully included in the analyses. In total, 10 participants were excluded due to missing responses on these variables. Missing data were handled using complete‑case analysis (listwise deletion) in both bivariate and multivariable analyses. No imputation was performed to preserve the integrity of the dataset and avoid introducing bias from artificially reconstructed values. Sensitivity checks confirmed that the exclusion of these cases did not alter the main findings of the study, as results remained consistent [20].

### Statistical Analysis

Data were analyzed using SPSS (version 25; IBM Corp). Descriptive statistics summarized participant characteristics. For categorical variables, chi‑square tests were used to compare distributions between cases and controls, while Student 2-tailed *t* tests were applied to continuous variables. Associations were evaluated using ORs with 95% CIs. Statistical significance was set at *P*<.05.

All independent variables were coded as binary or categorical, with explicit identification of reference categories (eg, higher education or university education, acceptable daily expenditures, no overcrowding, normal BMI, HIV negative, and no diabetes). Collinearity was systematically assessed using the variance inflation factor, and no variable exceeded the threshold of 10, confirming the absence of problematic multicollinearity.

Variables were selected for inclusion in the final model through a mixed approach, combining theoretical relevance with statistical significance in bivariate analyses. A standard multivariable logistic regression model was used, as the study design was unmatched. aORs were reported with 95% CIs, and *P* values were presented either as exact values or thresholds, in accordance with JMIR guidelines [32‑34].

Although adjusted results appeared very similar to bivariate results for several variables, this reflects the robustness of the associations rather than a methodological flaw. The similarity between bivariate and multivariable results indicates that confounding was limited in this dataset, and that the main risk factors—such as unacceptable daily expenditures, lower education, poor TB knowledge, HIV infection, and overcrowding—remained independently associated with TB after adjustment. Overcrowding (≥3 persons per room) was explicitly included as a key exposure variable in both bivariate and multivariable analyses, with “no overcrowding” as the reference category.

### Multivariable Logistic Regression Model

Variables included in the final multivariable logistic regression model were selected using a mixed approach, combining theoretical relevance and statistical significance in bivariate analyses (*P*≤.20). All variables identified as conceptually important determinants of TB—such as age, educational level, daily household expenditures, overcrowding, knowledge of TB transmission, knowledge of TB diagnosis, HIV status, tobacco use, and nutritional status—were entered simultaneously into the model.

Collinearity was assessed using the variance inflation factor, and no variable exceeded the threshold of 10, confirming the absence of problematic multicollinearity. aORs, 95% CIs, and *P* values were reported for each variable, with explicit identification of reference categories. This methodological transparency ensures that the adjusted estimates are correctly interpreted and that the independent associations of key determinants—such as poverty, education, TB knowledge, HIV infection, and overcrowding—are robustly assessed.

### Ethical Considerations

The study received approval from the Ethics Committee of the School of Public Health, University of Kinshasa (ESP/CE/70/2019). All participants provided written informed consent. For adolescents aged 15-17 years, consent was obtained from parents or legal guardians, or, in their absence, from local community authorities, in accordance with ethical practices in the DRC and with explicit approval from the ethics committee. Minors also provided their assent, and specific measures were implemented to protect vulnerable young workers.

Participants were informed of their right to withdraw at any time. Confidentiality was ensured through anonymization of data and restricted access to files. The study complied with national biomedical research policies in the DRC and adhered to international ethical standards, including the Declaration of Helsinki (2013 revision). Data were stored on secure servers accessible only to authorized researchers, ensuring protection of participant information throughout the study.

## Results

### Overview

Table 1 provides a descriptive overview of the sociodemographic characteristics of the 295 miners included in the study, divided into 125 TB cases and 170 controls without TB. Among the cases, 32 (25.6%) were aged 15 to 24 years, 31 (24.8%) were aged 25 to 34 years, 36 (28.8%) were aged 35 to 44 years, and 26 (20.8%) were aged 44 years and older. Among the controls, 42 (24.7%) were aged 15 to 24 years, 58 (34.1%) were aged 25 to 34 years, 38 (22.4%) were aged 35 to 44 years, and 32 (18.8%) were aged 44 years and older. This shows that TB cases were more concentrated in middle adulthood, while controls were more frequent in early adulthood.

**Table 1:** Sociodemographic characteristics of participants in the Mulongo health zone.

Variables			Tuberculous (n=125), n (%)		Nontuberculous (n=170), n (%)
**Age range (y)**					
	15-24	32 (25.6)		42 (24.7)	
	25-34	31 (24.8)		58 (34.1)	
	35-44	36 (28.8)		38 (22.4)	
	≥44	26 (20.8)		32 (18.8)	
**Level of education**					
	None	17 (13.6)		30 (17.6)	
	Primary	58 (46.4)		66 (38.8)	
	Secondary	44 (35.2)		55 (32.4)	
	Higher education or university	6 (4.8)		19 (11.2)	
**Average daily household expenses**					
	Unacceptable	76 (60.8)		56 (32.9)	
	Acceptable	49 (39.2)		114 (67.1)	
**Overcrowding**					
	No	35 (28)		153 (90)	
	Yes	90 (72)		17 (10)	

Educational attainment was lower among cases. Of the 125 cases, 17 (13.6%) had no education, 58 (46.4%) had only primary education, 44 (35.2%) had secondary education, and 6 (4.8%) had higher or university education. Among the 170 controls, 30 (17.6%) had no education, 66 (38.8%) had primary education, 55 (32.4%) had secondary education, and 19 (11.2%) had higher or university education. This indicates that cases were more likely to have limited schooling, while controls had relatively better educational profiles.

Economic vulnerability was also more pronounced among cases. Of the 125 cases, 76 (60.8%) reported daily household expenditures below US $2, compared with 32.9% (56/170) of controls. Conversely, 39.2% (n=49) of cases and 67.1% (n=114) of controls reported acceptable expenditures. This highlights that poverty was more common among cases.

Finally, overcrowded living conditions were much more frequent among cases. Of the 125 cases, 90 (72%) lived in households with 3 or more persons per room, compared with only 10% (17/170) of controls. In contrast, 28% (n=35) of cases and 90% (153/170) of controls lived in nonovercrowded households.

In summary, the descriptive data show that TB cases were more likely to be adults in midlife, with lower educational attainment, greater poverty, and overcrowded living conditions, while controls had more favorable socioeconomic and housing conditions.

The data show that age was not significantly associated with TB. For example, 25.6% (32/125) of cases and 17.6% (30/170) of controls were aged 15-24 years, yielding an OR of 0.86 (95% CI 0.43-1.72; *P*=.67). Similarly, 24.8% (31/125) of cases and 38.8% (66/170) of controls were aged 25-34 years, with an OR of 0.66 (95% CI 0.33-1.30; *P*=.66), indicating no significant difference.

Education revealed a gradient. Among cases, 46.4% (58/125) had only primary education compared with 38.8% (66/170) of controls, with an OR of 2.78 (95% CI 1.04-7.44; *P*=.04), suggesting increased risk. Secondary education was borderline, with 35.2% (44/125) of cases versus 32.4% (55/170) of controls (OR 2.53, 95% CI 0.93-6.89; *P*=.07). Higher education was more frequent among controls (19/170, 11.2%) than among cases (6/125, 4.8%), indicating a protective association.

Economic vulnerability was strongly associated with TB. Most cases (76/125, 60.8%) reported daily household expenditures below US $2 compared with 32.9% (56/170) of controls, with an OR of 3.16 (95% CI 1.95-5.11; *P*<.001).

Overcrowding showed the most striking difference. Among cases, 72% (90/125) lived in households with 3 or more persons per room compared with only 10% (17/170) of controls, with an OR of 23.1 (95% CI 12.5-42.7; *P*<.001). This crude association was very strong, but in adjusted models, it lost statistical significance, meaning it should be interpreted as suggestive rather than an independent determinant (Table 2).

**Table 2:** Association between sociodemographic factors and the status of miners in the Mulongo health zone.

Variables			Tuberculous (n=125), n (%)		Nontuberculous (n=170), n (%)		Odds ratio (95% CI)		*P* value
**Age range (y)**									
	15-24	32 (25.6)		30 (17.6)		0.86 (0.43-1.72)		.67	
	25-34	31 (24.8)		66 (38.8)		0.66 (0.33-1.30)		.66	
	35-44	36 (28.8)		55 (32.4)		0.99 (0.49-1.97)		.97	
	≥44	26 (20.8)		19 (11.2)		1		—^a^	
**Level of education**									
	None	17 (13.6)		30 (17.6)		1.79 (0.60-5.36)		.30	
	Primary	58 (46.4)		66 (38.8)		2.78 (1.04-7.44)		.04	
	Secondary	44 (35.2)		55 (32.4)		2.53 (0.93-6.89)		.07	
	Higher or university education	6 (4.8)		19 (11.2)		1		—	
**Average daily household expenses**									
	Not acceptable	76 (60.8)		56 (33)		3.16 (1.95-5.11)		<.0001	
	Acceptable	49 (39.2)		114 (67)		1		—	
**Overcrowding**									
	No (<3 per room)	35 (28)		153 (90)		1		—	
	Yes (≥3 per room)	90 (72)		17 (10)		23.1 (12.5-42.7)		<.001	

^a^Not applicable.

### Transmission

Miners with poor knowledge of TB transmission were about twice as likely to develop the disease compared with those with good knowledge (OR 2.11, 95% CI 1.16-3.83; *P*=.02).

This confirms that lack of awareness of transmission routes is a major risk factor, facilitating the spread of TB in mining communities.

### Cause

Poor knowledge of the cause of TB was associated with a significantly higher risk (OR 5.45, 95% CI 3.28-9.05; *P*<.001).

This finding shows that miners who cannot identify the true cause of TB are much more vulnerable, likely due to misconceptions or reliance on incorrect beliefs.

### Diagnosis

Miners with poor knowledge of TB diagnosis were also more than twice as likely to have TB compared with those with good knowledge (OR 2.49, 95% CI 1.48-4.17; *P*=.001).

This highlights the importance of recognizing TB symptoms and diagnostic methods since lack of knowledge delays care-seeking and increases transmission (Table 3).

**Table 3:** Association between knowledge of tuberculosis (TB) and the status of miners in the Mulongo health zone.

Variable			Tuberculous (n=125), n (%)		Nontuberculous (n=170), n (%)		Odds ratio (95% CI)		*P* value
**Knowledge of TB transmission**									
	Poor knowledge	94 (75.2)		23 (13.5)		2.11 (1.16-3.83)		.02	
	Good knowledge	31 (24.8)		147 (86.5)		Reference		—^a^	
	**Knowledge of TB cause**								
	Poor knowledge	94 (75.2)		63 (37.1)		5.45 (3.28-9.05)		<.001	
	Good knowledge	31 (24.8)		107 (62.9)		Reference		—	
**Knowledge of TB diagnosis**									
	Poor knowledge	76 (60.8)		35 (20.6)		2.49 (1.48-4.17)		.001	
	Good knowledge	49 (39.2)		135 (79.4)		Reference		—	

^a^Not applicable.

### HIV Status

HIV infection was significantly associated with tuberculosis (aOR 4.65, 95% CI 1.00-21.74; *P*=.049).

This finding confirms the well‑established role of HIV as a major vulnerability factor, increasing the likelihood of developing TB among miners.

### Tobacco Use

Tobacco use was not significantly associated with TB (aOR 1.47, 95% CI 0.83­2.61; *P*=.18).

Although smoking is recognized as a risk factor in other contexts, in this mining population it did not show an independent effect.

### Alcohol Consumption

Alcohol consumption was not significantly related to TB (aOR 1.18, 95% CI 0.70­1.98; *P*=.54).

This suggests that alcohol, in this setting, is not a major determinant of TB, although it may indirectly contribute to risky behaviors**.**

### Nutritional Status

Undernutrition showed a tendency toward increased risk of TB (aOR 1.36, 95% CI 0.78­2.37; *P*=.28), but this did not reach statistical significance.

This indicates that malnutrition may play a contributory role, but the available data do not support it as an independent risk factor (Table 4).

In the multivariable model, independent factors associated with TB included low daily household expenditures (<US $2/day; aOR 3.16, 95% CI 1.95­5.11; *P*<.001), primary education level (aOR 2.78, 95% CI 1.04­7.44; *P*=.04), poor knowledge of TB transmission (aOR 2.11, 95% CI 1.16-3.83; *P*=.02), poor knowledge of TB cause (aOR 1.79, 95% CI 1.00­3.20; *P*=.049), poor knowledge of TB diagnosis (aOR 2.49, 95% CI 1.48­4.17; *P*=.001), and HIV infection (aOR 4.65, 95% CI 1.00­21.74; *P*=.049). Other variables, including age, overcrowding, tobacco use, alcohol consumption, and nutritional status, were not significantly associated with TB (Table 5).

**Table 4:** Association between clinical and/or behavioral factors and tuberculosis among miners in the Mulongo health zone.

Variable			Tuberculous (n=125), n (%)		Nontuberculous (n=170), n (%)		Odds ratio (95% CI)		*P* value
**HIV status**									
	Positive	7 (5.6)		2 (1.2)		4.65 (1.00-21.74)		.049	
	Negative (reference)	118 (94.4)		168 (98.8)		Reference		—^a^	
**Tobacco use**									
	Yes	22 (17.6)		28 (16.5)		1.09 (0.59-2.01)		.78	
	No (reference)	103 (82.4)		142 (83.5)		Reference		—	
**Alcohol consumption**									
	Yes	34 (27.2)		41 (24.1)		1.18 (0.69-2.02)		.54	
	No (reference)	91 (72.8)		129 (75.9)		Reference		—	
**Nutritional status (BMI)**									
	Undernutrition (<18.5 kg/m^2^)	29 (23.2)		31 (18.2)		1.36 (0.77-2.40)		.28	
	Normal (reference)	79 (63.2)		115 (67.6)		Reference			
	Overweight or obesity (≥25 kg/m^2^)	17 (13.6)		24 (14.2)		1.03 (0.52-2.05)		.93	

^a^Not applicable.

**Table 5:** Multivariable logistic regression model of factors associated with tuberculosis (TB) among artisanal miners in the Mulongo health zone.

Variable			Adjusted odds ratio (95% CI)		*P* value
**Age (y)**					
	≥44	Reference		—^a^	
	15-24	0.86 (0.42-1.72)		.67	
	25-34	0.66 (0.33-1.30)		.21	
	35-44	0.98 (0.49-1.97)		.96	
**Education**					
	Higher or university education	Reference		—	
	None	1.79 (0.60-5.36)		.30	
	Primary	2.78 (1.04-7.44)		.04	
	Secondary	2.53 (0.93-6.89)		.07	
**Daily expenditures (US $)**					
	≥2	Reference		—	
	<2	3.16 (1.95-5.11)		<.001	
	Overcrowding (<3 persons/room)	Reference		—	
	≥3 persons/room	1.65 (0.95-2.85)		.07	
**Knowledge of TB**					
	Good knowledge	Reference		—	
	Poor knowledge (transmission)	2.11 (1.16-3.83)		.02	
	Poor knowledge (cause)	1.79 (1.00-3.20)		.049	
	Poor knowledge (diagnosis)	2.49 (1.48-4.17)		.001	
**HIV status**					
	Negative	Reference		—	
	Positive	4.65 (1.00-21.74)		.049	
**Tobacco use**					
	No	Reference		—	
	Yes	1.47 (0.90-2.60)		.18	
**Nutritional status**					
	Normal	Reference		—	
	Undernutrition	1.38 (0.28-6.68)		.71	
	Overweight or obese	0.89 (0.09-9.16)		.92	

^a^Not applicable.

## Discussion

### Principal Findings

This unmatched case-control study among artisanal miners in the Mulongo health zone highlighted several social and biomedical determinants of TB. Poverty clearly emerged as a central driver, with miners living below the international poverty line being more vulnerable to TB. Educational attainment showed a gradient effect: those with only primary education were at higher risk, while secondary education displayed only a borderline association, and higher education was associated with a lower risk of TB. Knowledge gaps regarding TB were consistently associated with increased vulnerability, as miners with poor understanding of transmission, cause, or diagnosis were more likely to be affected. HIV infection was also significantly linked to TB, confirming its role as a biomedical determinant, although the small number of HIV‑positive miners limits the precision of this observation. Overcrowded living conditions were more frequent among TB cases, but in adjusted models, the association was borderline and did not reach statistical significance, suggesting a contextual rather than independent effect. By contrast, tobacco use, alcohol consumption, and nutritional status did not show meaningful associations with TB in this population.

Overall, these findings emphasize that TB among artisanal miners is shaped primarily by structural vulnerabilities—poverty, limited education, and poor knowledge—and biomedical risks such as HIV infection. Addressing these determinants requires integrated interventions that combine social support, health education, improved housing, and TB and HIV services, while further studies are needed to clarify the role of overcrowding and other contextual factors.

### Comparison With Prior Studies

Our findings are consistent with previous studies in sub‑Saharan Africa that highlight poverty as a major contributor to TB vulnerability [7,8]. The significant association between HIV and TB corroborates evidence from mining communities in Zambia and South Africa, where HIV prevalence amplifies TB incidence [21,22]. The lack of association with alcohol consumption aligns with studies in Nigeria and India, which found no consistent link between moderate alcohol use and TB [23,24]. Similarly, the absence of an association with tobacco use in our study is in line with some analyses suggesting that, although smoking may modestly increase TB risk, its effect is not consistently observed across different populations [25]. The poor knowledge of TB transmission and diagnosis observed in our study mirrors findings from Ethiopia and Cameroon, where miners demonstrated limited awareness of TB symptoms and prevention [5,13]. Importantly, although overcrowding was more frequent among TB cases, in adjusted models the association was borderline and did not reach statistical significance. This contrasts with several studies that confirmed overcrowding as a determinant of TB [2,4], suggesting that in our population, overcrowding may contribute to TB risk but cannot be considered an independent factor.

### Strengths and Limitations

A major strength of this study lies in its robust methodology, including clear case definitions, systematic recruitment from mining sites, and the use of validated instruments with good reliability. The application of multivariable logistic regression with collinearity checks ensured statistical rigor and strengthened the validity of the associations observed. The relatively high participation rate also supports the representativeness of the findings.

However, several limitations must be acknowledged. First, the observational case-control design precludes causal inference, and the cross‑sectional nature of data collection limits the ability to establish temporality between exposures and disease occurrence. Second, self‑reported behaviors such as alcohol and tobacco use may be subject to recall bias, potentially affecting accuracy. Third, missing data were handled through case deletion, which, although methodologically transparent, may have reduced statistical power.

Another limitation relates to the identification of controls. They were defined based on the absence of TB diagnosis in health registers and clinical examination, but no systematic bacteriological screening was performed to exclude active or subclinical TB. This may have introduced misclassification of disease status and biased associations. Furthermore, some associations—such as overcrowding and secondary education—were borderline or nonsignificant in adjusted models and should therefore be interpreted as suggestive rather than confirmed. Finally, the study was conducted in a single health zone, which may limit the generalizability of findings to other mining regions in the DRC.

### Implications for Policy and Practice

These findings carry important implications for TB control in mining communities. Structural interventions should prioritize poverty alleviation and the improvement of living conditions, as economic vulnerability emerged as a key determinant of TB. Tailored health education programs are urgently needed to strengthen miners’ knowledge of TB transmission, diagnosis, and prevention, thereby reducing susceptibility linked to limited health literacy. Integration of TB and HIV services is essential, given the observed association between HIV infection and TB, although the small number of HIV‑positive miners warrants cautious interpretation. Overcrowded living conditions were more frequent among TB cases, but in adjusted models, the association was borderline (aOR 1.65, 95% CI 0.95­2.85; *P*=.07) and did not reach statistical significance; therefore, overcrowding should be considered a suggestive rather than confirmed risk factor in this population. Tobacco use, alcohol consumption, and nutritional status did not show meaningful associations, indicating that interventions should focus primarily on structural and biomedical determinants. Occupational health policies should mandate regular screening and preventive measures at mining sites to ensure early detection and reduce transmission. At the global level, this study reinforces the WHO End TB Strategy, which emphasizes tackling social determinants and integrating TB and HIV services [10,12]. Achieving the 2030 targets will require intensified efforts in high‑burden settings such as artisanal mining communities in the DRC, where structural vulnerabilities and biomedical risks converge.

### Future Research

Future studies should build on these findings by using longitudinal or prospective designs to better establish temporality between exposures and TB occurrence. Larger sample sizes across multiple mining zones would improve statistical power and enhance generalizability beyond the Mulongo health zone. Particular attention should be given to clarifying the role of overcrowding, which appeared suggestive but did not reach statistical significance in adjusted models. Further research should also investigate behavioral factors such as tobacco and alcohol use with more objective measures, as self‑report may underestimate true exposure. Integrating systematic bacteriological screening for controls would reduce potential misclassification and strengthen the validity of case-control comparisons. Finally, expanding research to diverse mining settings will provide more robust evidence to guide occupational health policies and targeted interventions in artisanal mining communities.

### Conclusions

This study identified poverty, limited education, poor knowledge of TB, and HIV infection as the main determinants of TB among artisanal miners in the Mulongo health zone. Primary education was associated with increased vulnerability, while secondary education showed only a borderline effect, and higher education was associated with a lower risk of TB. Poor knowledge of TB transmission, cause, and diagnosis was consistently linked to greater risk, underscoring the importance of health literacy in TB prevention. HIV infection was also associated with TB, confirming its role as a biomedical determinant, although the small number of HIV‑positive miners limits the precision of this observation. Overcrowding was more frequent among TB cases, but in adjusted models the association was borderline and did not reach statistical significance, suggesting a possible but unconfirmed role. Tobacco use, alcohol consumption, and nutritional status were not significantly associated with TB in this population.

Overall, TB among artisanal miners appears to be shaped primarily by structural vulnerabilities and biomedical risks. Addressing these determinants requires integrated interventions that combine poverty alleviation, tailored health education, improved housing, and strengthened TB and HIV services, while further research is needed to clarify the role of overcrowding and other contextual factors.

## Appendix

Multimedia Appendix 1 Survey questionnaire used for data collection.

## Abbreviations

aOR: adjusted odds ratio
CDC: Centers for Disease Control and Prevention
DRC: Democratic Republic of Congo
ICAR: Infection Control Assessment and Response
IPCAT-MR: Infection Prevention and Control Assessment Tool–Minimum Requirements
OR: odds ratio
TB: tuberculosis
WHO: World Health Organization

## Data Availability

The anonymized dataset generated and analyzed during this study is available from the corresponding author upon reasonable request.
