# Supplementary material for: Tuberculosis Among Artisanal Miners in the Mulongo Health Zone, Democratic Republic of Congo: Unmatched Case-Control Study
Source: JMIR Form Res. 2026 Oct 19;10:e93635. doi: 10.2196/93635 (PMC13728770; doi:10.2196/93635)
Supplement: Supplementary file 1 [file formative_v10i1e93635_app1.docx]

**APPENDICES**

**DEMOCRATIC REPUBLIC OF THE CONGO**

UNIVERSITY OF KINSHASA

SCHOOL OF PUBLIC HEALTH

**TRAINING PROGRAM IN FIELD EPIDEMIOLOGY AND LABORATORY MANAGEMENT**

**SURVEY QUESTIONNAIRE FOR ARTISANAL MINERS**

***“Factors associated with tuberculosis among artisanal miners in the Mulongo health zone, Haut-Lomami province”***

**Section 1: Identification**

001 Questionnaire Number /_/_/_/_/

002 District_____________________________ /_/_/

003 Avenue/Street______________________________

004 Investigator: Code /_/_/ Name______________________________

005 Date of investigation: /_/_/ /_/_/ /_/_/

006 Miner's Status: Tuberculous Yes No

007 Supervised by: Name______________________________ Date /_/_/ /_/_/ /_/_/

009 Supervisor's signature _________________________

**Section 2: Sociodemographic characteristics**

| **No.** | **FILTER QUESTION** | **ANSWERS-CODES** |
| --- | --- | --- |
| Q100 | Time the investigation began | /_/_/Hour /_/_/minutes |
| Q101 | In what month and year were you born? | Month /_/_/  I don't know the month of **98**  No response **99**  Year /_/_/  I don't know the year **98**  No response **99** |
| Q102 | How old are you now? (Compare and correct if necessary based on Q101) | Age in completed years **/_/_/** |
| Q103 | Weight (kg) | **/_/_/_/_/** |
| Q104 | Size (cm²) | **/_/_/** |
| Q105 | What is the highest level of education you have achieved? | None **1**  Primary **2**  Secondary **3**  Higher/University **4** |
| **2.1 Knowledge of tuberculosis** | | |
| Q106 | Can you tell us what causes tuberculosis? | _1: know  0: Don't know |
| Q107 | What are the signs of tuberculosis? | Cough **1**  Fever **2**  Night sweats **3**  Weight loss **4**  Asthenia **5**  Anorexia **6** |
| Q108 | How is tuberculosis transmitted? | Concentration of pffluge droplets **1**  The time taken to breathe contaminated air **2**  Living in the same house as a sick person who has not started their treatment **3**  Other details to be specified __________ **4** |
| Q109 | How is tuberculosis diagnosed? | **1 knows**  **0 doesn't know** |
| Q110 | How much do you spend on average per day on your household needs? | **/_/_/_/$**  **1 less than $1.25**  **2 over $1.25** |
| Q111 | How many people live in your household? | /_/ people  1 less than or equal to 2 people  2 or more people |
| **2.2 Risky Behavior** | | |
| Q112 | Do you consume alcohol? | Yes **1**  No. **2** |
| Q113 | Do you use tobacco? | Yes **1**  No. **2** |
| **2.3 Factors related to the patient's condition** | | |
| Q114 | Are you diabetic? | Yes **1**  No **0** |
| Q115 | Have you ever been tested for HIV? | Yes **1**  No **0** |
| Q116 | What was the result? | Positive **1**  Negative **2** |

THANK YOU

DEMOCRATIC REPUBLIC OF THE CONGO

UNIVERSITY OF KINSHASA

SCHOOL OF PUBLIC HEALTH

TRAINING PROGRAM IN FIELD EPIDEMIOLOGY AND LABORATORY MANAGEMENT

"Factors associated with tuberculosis among artisanal miners"

INFORMED CONSENT

Welcome. My name is ___________________________, and I work with the Mulongo Health Zone. We are conducting a study on factors associated with tuberculosis among artisanal miners. We would appreciate your participation in this study. The information you provide will help us plan and improve health services. The interview will take approximately 20 minutes. All information provided will be kept confidential and will not be shared with anyone.

Your participation in this study is voluntary, and you may choose not to answer any individual questions or any questions at all. However, we would like you to participate in this study as your views are important.

Now, do you have any questions about this study?

Do you agree to participate in this study? YES NO

THANK YOU
